# Addressing Occupational Dysfunction via Telehealth: A Scoping Review

**DOI:** 10.5195/ijt.2024.6638

**Published:** 2025-01-15

**Authors:** James T. Foster, Mary Zadnik

**Affiliations:** 1 Master of Occupational Therapy Program, Messiah University, Mechanicsburg, PA, USA; 2 Occupational Therapy Program, St. Edward's University, Austin, TX, USA

**Keywords:** Occupational dysfunction, Occupational therapy, Telehealth

## Abstract

**Background:**

This review aims to identify how telehealth was utilized to address occupational dysfunction during the COVID-19 pandemic period.

**Methods:**

A scoping review following Arksey and O'Malley's stages was utilized to explore appropriate research. The scoping review covered articles from January 2020 to December 2022.

**Results:**

A total of 23 articles are included in this scoping review which include 15 from the adult age group and eight from the pediatric.

**Conclusions:**

The use of telehealth during the COVID-19 pandemic has been shown to improve occupational dysfunction, both within the adult and pediatric settings. Further research is needed to demonstrate the effectiveness of the use of telehealth to address occupational dysfunction.

Occupational therapy (OT) is a health discipline that utilizes everyday life occupations to address dysfunction and or disabilities with persons, groups, or populations (American Occupational Therapy Association [AOTA], 2020). The term “telehealth” is utilized in a wide variety of contexts, but for the purpose of this paper, the authors are defining telehealth as a service delivery model that utilizes electronic hardware and software, which allows clients and providers to communicate via sound and sight, to deliver occupation-based services to individuals, groups, and populations. Over the past twenty years, occupational therapy has acknowledged the opportunities to provide services through telehealth (AOTA., 2018).

The COVID-19 pandemic had a significant impact on occupational therapy, other rehabilitation disciplines, and the ability to deliver essential services. In many settings, there were modifications or decreased access to occupational therapy, that were due to local, state, or nationwide restrictions (Hoel et al., 2021). These restrictions had a negative impact on individuals, groups, and the population's ability to engage in typical and necessary occupations of their everyday lives (White et al., 2021). Occupational therapy service delivery was preserved using telehealth technology, which met the needs of individual clients, groups, and populations (Hoel et al., 2021).

During the COVID-19 pandemic, the use of telehealth allowed occupational therapy practitioners to improve their efficiency, decrease therapy delays and missed appointments due to sickness by the therapist and the clients (Bopp, 2022). The delivery of rehabilitation services via telehealth was endorsed by the Centers for Medicare & Medicaid Services (2020) as an acceptable delivery method for such services as occupational therapy, physical therapy, and speech therapy. Ng and Park (2021) found that 81.2 % of Medicare beneficiaries received services that were offered through telehealth during the COVID-19 pandemic.

Cason (2014) explored telehealth as a “rapidly developing service delivery model” and acknowledged that “telehealth can improve access to occupational therapy services and facilitate interprofessional collaboration” (p 33). There were also multiple pre-COVID-19 studies which revealed that the telehealth setting may have the same or better outcomes as in-person delivery (Kinley et al., 2012; Taber-Dougherty et al., 2010). Kinley et al. (2012) found a higher outcome of smoking cessation in adults with mental health disorders who were seen via telehealth as compared to in-person services. Taber-Doughty (2010) found that in adults with intellectual disabilities, their ability to accomplish novel household activities with independence was greater in those who received services via telehealth compared to in-person delivery.

Previous research suggested that successful introduction and development of telehealth within a health care system required a 23-month time frame for implementation (Hare et al., 2020). This time frame was not afforded to occupational therapy practitioners, nor the world, at the start of the COVID-19 pandemic. Instead, practitioners were put in a position to develop telehealth delivery models within days or weeks to minimize interruptions in services. Wosik et al. (2020) described three phases of telehealth implementation within the medical community as it transitioned from the “stay at home” phase, to the “inpatient COVID-19 related surge” phase and finally to the “post pandemic recovery” phase. This final phase is characterized by healthcare systems having improved long-term telehealth infrastructure. Bolden (2022) notes that “most” allied health professionals reported being “somewhat prepared” to transition their practice to telehealth delivery as compared to the “few” who reported being “very prepared” or “not at all prepared” for the transition.

The need for the American Occupational Therapy Association to support waivers to charge for occupational therapy services was identified early within the pandemic (AOTA, 2021). This allowed flexibility and guidance to support practitioners as they negotiated their way through use of this new technology. The ability of occupational therapy practitioners to provide services in-person or via telehealth allowed patients and families to select the rehabilitation delivery method that worked best for them (Robinson et al., 2021).

A full understanding of which settings occupational therapy practitioners utilized telehealth throughout the COVID-19 pandemic, and its effectiveness, is still emerging. Prior to the pandemic, the World Federation of Occupational Therapy (2017) had identified the investigation of telehealth delivery as one of its international research priorities. For that reason, a scoping review of the recent (since 2020) literature that utilized the use of telehealth to address occupational dysfunction in groups and populations is meaningful. It will serve to identify populations where occupational therapy practitioners utilized telehealth to accomplish pre-established goals. This will benefit practitioners, at-risk groups, and populations during future worldwide medical emergencies. To that end, the identified research question that this scoping survey aspired to answer was: *How is occupational dysfunction addressed via telehealth?* Relevant studies presented within this review include those that were published since 2020, which incorporate the COVID-19 period.

There have been previous scoping and systemic reviews of the use of telehealth in occupational therapy that pre-date the COVID-19 pandemic era. Nobakht et al. (2017) completed a scoping review that explored the extent of its usage and the types of technologies commonly used and reviewed research from 1990 to 2015. Önal et al. (2021) completed a scoping review in pediatric occupational therapy exploring access of services in articles published from 2000 to 2020. Albritton et al. (2021) performed a scoping review that explored the use of telehealth by occupational therapists to address chronic disease self-management. Patterson et al. (2021) completed a systematic review of patient satisfaction with telehealth, specifically within rural settings, and found four articles that noted “high satisfaction” for patients using telehealth for rehabilitation which occurred across various diagnoses, age groups, and types of technology utilized. In addition, professionals’ manners, quality of telehealth equipment, and access to health care practitioners were all reported to be satisfactory.

## Methods

This scoping review was structured by the authors to match the Arksey and O'Malley (2005) framework which provides five specified steps of the review to include: identifying the research question, identifying relevant studies, study selection, data charting and finally, collating, summarizing, and reporting the results. Scoping review is an increasingly utilized way to collect and share a body of evidence, while not seeking to assess the quality of the evidence (Armstrong et al., 2011).

Additional rehabilitation disciplines have recently demonstrated efficacy in the delivery of their interventions via telehealth. Malandraki et al. (2021) demonstrated efficacy in the use of a telehealth service delivery model for dysphagia treatment across the lifespan. McGill and Schroth (2022) highlighted a reduction in stuttering in an adult who received speech therapy services via telehealth. Horton et al. (2021) found no significant differences in pre to postoperative changes in outcomes for patients post hip or knee arthroplasty in a population that received traditional physical therapy compared to those who received their physical therapy via telehealth. In addition, physical therapy delivered via telehealth for low-back pain, hip and knee replacement, and multiple sclerosis was found to be comparable with in-person rehabilitation or better than lack of rehabilitation (Seron et al., 2021). Counseling for individuals with a medication for opioid use disorder, delivered through telehealth, was effective and highlighted multiple benefits such as ensured continuity and protection of the health of the patient (Hughton et al., 2021).

### Identified Relevant Studies

The terms used to search for relevant studies included “telehealth” and “occupational therapy” within CINAHL and MEDLINE. The characteristics of each included article's characteristics were those that explored occupational dysfunction that were addressed via telehealth, plus the goal(s) achieved within the setting. Each utilized article was organized together with similar populations served. Included articles were also explored for their references that might add depth to the review.

### Study Selection

The review period includes studies published starting in 2020 which coincides with the COVID-19 pandemic time frame. The studies included are those that explored the use of telehealth as a service delivery method to address occupational dysfunction. All the studies included were peer-reviewed and from academic journals. The search for articles netted 1,709 articles, of which twenty-three were included. (Table 1). Only peer reviewed articles were included and all articles that presented actual clinical outcomes were included, which excluded articles about therapists and patients’ perceptions, operational creation of programs and other scoping or systematic reviews.

**Table 1:** Search Process of Academic Journals within Academic Institution Library (CINAHL and Medline).

Initial search results= 1,709
↓
References removed due to duplicate, or “perception” articles= 1,505
↓
References removed due to “systematic” or “scoping” reviews= 28
↓
References removed due to lack of clinical outcomes= 154
↓
Initial scoping review articles= 22
↓
138 references screened of 22 original articles netted inclusion of 1 article.

*Note*. Search Words: “Telehealth and Occupational Therapy” Time Frame 2020–2023 and Peer Reviewed and Academic Journals within Academic Institution Library (CINAHL and Medline).

A total of twenty-three (23) studies that were published between 2020 and 2023 were included in this study. Fifteen studies are within the adult occupational therapy age group and eight within the pediatric population (See Table 2). Evidence found within each study is presented below.

**Table 2:** Study Populations

Total:			23
Pediatric populations			8 (35%)
	Health outcomes	Burns, diabetes, general health care	5 (22%)
	Autism		2 (9%)
	School based	Writing	1 (4%)
Adult populations			15 (65%)
	Community based	Home bound, older adults	4 (17%)
	Neurological	Stroke, MND	3 (13%)
	General medical	Cancer, COVID	3 (13%)
	Emotional	Developmental disabilities, wellness	3 (13%)
	Work or upper extremity	Return to work, upper extremity rehab	2 (9%)

## Discussion

### Pediatric Telehealth Review

Jacobs et al. (2021) presented a feasible method to treat midline crossing deficits in children via the use of games delivered within a telehealth system. The TeleWrite handwriting system was used to assess handwriting in third graders via telehealth and was noted to have strong interrater reliability and validity (Guzman & Grajo, 2021). Parents of children with special healthcare needs completed the Telehealth Usability Questionnaire and reported that occupation-based education delivered via telehealth was “effective and useful” (Smith et al., 2022). Phillips et al. (2022) highlighted the use of telehealth for children who received post-surgical outpatient burn occupational therapy services and confirmed the ability to deliver complicated rehab to rural and remote children after burn injuries. Jewell et al. (2022) noted “met health goals” for Type 1 diabetic children who participated in an occupation-based telehealth program.

There were several studies that more recently highlighted the use of telehealth within the population of children with autism spectrum disorder (ASD) (Fernandes et al., 2022; Hawkins et al., 2022; Tanner et al., 2020). Fernandes et al. (2022) presented the successful telemonitoring of children and adolescents with ASD, for the purpose of providing interventions related to daily schedules, activities of daily living, and social communication. Pediatric subjects on the ASD with feeding difficulties demonstrated increased oral acceptance after participating in telehealth sessions (Hawkins et al., 2022. Tanner et al. (2020) reported “needs being met” and high satisfaction rates (98.97%) of parents whose children received therapy services via telehealth.

### Adult Telehealth Review

Home and community settings have been studied for the use of interventions delivered via telehealth to adults (Boone et al., 2022; Moyers Cleveland et al., 2022; Schepens-Niemiec et al., 2021; Zahoransky & Lape, 2020). Zahoransky and Lape (2020) found statistically significant improvements in clients’ perception of performance and satisfaction with activities of daily living performance when services were provided via a hybrid model, including telehealth, to homebound clients. Boone et al. (2022) studied the delivery of the Activity Card Sort (ACS3) to community dwelling adults via telehealth and findings suggest that the ACS3 can be administered virtually with good validity compared to in-person delivery. To address loneliness and isolation that occurred during COVID-19, meaningful music activities were delivered to older adults via telehealth and were found to change roles and habits that subsequently improved occupational participation (Moyers-Cleveland et al., 2022). Schepens-Niemiec et al. (2021) studied long term lifestyle interventions that were delivered through a mix of telehealth and in-person services for Latino adults and found significant improvements in blood pressure, social roles, and activity satisfaction.

Populations with occupational dysfunction related to neurologic conditions have used telehealth as well (Ghosh & Cox, 2021; Grampurohit et al., 2022; Laver et al., 2020). Ghosh and Cox (2021) found telehealth to be effective to assess persons with motor neuron diseases. A stroke patient who received occupational post-discharge telehealth services experienced no readmission and showed improved quality of life, mood, and participation on the Stoke Impact Scale (Grampurohit et al., 2022). Laver et al. (2020) found no difference in activity of daily living performance in stroke survivors between those who received post hospital discharge instructions via telehealth versus those who received in-person education.

Cancer and general medicine populations have been shown to successfully receive occupation-based services via telehealth (Gilboa et al., 2021; Rubio et al., 2022; Sclarsky & Kumar, 2021). Cognitive related deficits impact the lives of millions of people worldwide, with up to 75% of cancer survivors experiencing cognitive impairments (Gilboa et al., 2021). The use of a telehealth service delivery model was shown to improve occupational performance, cognitive functioning, and quality of life within this population (Gilboa et al., 2021). This demonstrates the benefit of telehealth when addressing cognitive deficits and everyday life skills for cancer survivors. Rubio et al. (2022) studied its use with women who survived breast cancer with related lymphedema. The study showed that telehealth services which were focused on the Remotivation Process resulted in women who had increased awareness of self-management programs and occupational performance (Rubio et al., 2022). In addition, a case study was presented in which telehealth services were provided to two seniors with COVID-19 and demonstrated clinically significant changes in valued areas of occupation, as shown on the Canadian Occupational Performance Measure (Sclarsky & Kumar, 2021).

Adults with social and emotional deficits have accessed occupational therapy services through telehealth (Benham et al., 2022; Connor et al., 2021; Sánchez-Guarnido et al., 2021). Benham et al. (2022) noted significant changes in sleep quality and perceived stress in college students who attended telehealth mindfulness sessions. Connor et al. (2021) compared soft skills in young adults with autism within social groups, and hybrid versus in-person. They found increased confidence in soft skill communication in the hybrid (including telehealth) group versus in-person treatment sessions. Sánchez-Guarnido et al., (2021) found that mental health patients who received occupational therapy services via telehealth relapsed less in the following six months compared to those who did not.

An additional population whose occupational dysfunction can be addressed through telehealth are adults who are attempting to return to work and improve upper extremity function. Gross et al. (2021) studied the ability of Canadian compensation workers to return to work. This group reported successful completion of rehabilitation programs, although the delivery of services occurred via a telehealth delivery method. Harper et al. (2022) found that telehealth delivered to outpatient hand and upper limb netted similar outcomes and significantly fewer withdrawals from therapy.

The review revealed that 17 studies delivered occupational therapy services strictly through telehealth, while six used a hybrid approach combining telehealth and in-person sessions. All nine occupations within the Occupational Therapy Practice Framework 4^th^ Edition (AOTA, 2020) are noted to be addressed within the articles surveyed. Studies using strict telehealth interventions often focused on narrow aspects of occupational therapy, especially in pediatric populations, such as handwriting assessment, midline crossing, or social skills for children with ASD. These findings indicate telehealth's effectiveness in delivering targeted, focused interventions that can be addressed remotely. Hybrid studies were more frequently applied to adult populations with complex needs, such as return-to-work programs and chronic lifestyle management. This division suggests that hybrid models may be better suited for complex or multifaceted cases, allowing for a balance between remote and hands-on in-person care.

Eighteen of the studies reviewed were conducted during the COVID-19 pandemic, indicating an urgent shift to telehealth as a primary means of service delivery. Only three studies pre-dated the pandemic, while two occurred afterward. Notably, all pediatric studies were conducted during the pandemic, highlighting telehealth's ability to rapidly adapt to meet pediatric care needs in restrictive conditions. In adult populations, there has been continued use of telehealth beyond the pandemic, especially through hybrid models for managing chronic conditions and complex rehabilitation. This timeline illustrates telehealth's growth during a time of need and urgency, while hybrid models are emerging as a sustainable approach for the future of occupational therapy in complex adult cases.

While this scoping survey highlights the benefits of telehealth for occupational therapy practitioners, two of the articles also identify weaknesses or decreased effectiveness of the service delivery model. Laver et al., (2020) found telehealth useful for providing education, however the service delivery model did not result in significant improvements in daily living activities compared to traditional in-person methods. Moyers-Cleveland et al. (2022) highlighted the positive occupational participation, but the study also found that some participants struggled with the technology aspect, which limited effectiveness for some participants.

## Limitations

The sample size of twenty-three (N=23), limits statistical power, and ability to apply findings broadly. It also did not include articles that documented therapists’ perceptions or included operational instructions or challenges with creating telehealth programs. Several of the articles were completed in specific or single geographic locations which reduces the applicability of findings to diverse or underserved populations. The lack of control groups in most studies make it challenging to attribute outcomes solely to telehealth or hybrid interventions.

## Conclusion

This scoping review highlights the increasing use of telehealth by occupational therapy practitioners across various settings to address occupational dysfunction. Despite the growing implementation of telehealth, further research is necessary to establish its long-term efficacy. Technological barriers can be related to digital literacy divide between younger and older demographics of our population (Robinson-Bert et al., 2020). Clinician bias, wherein some therapists believe in-person delivery is inherently more effective than virtual delivery, plus lack of training with telehealth that incorporates intervention planning and ability to develop rapport, also influences its integration (Feldhacker et al., 2022).

Despite these barriers, the positive outcomes reported in this review call for a reassessment of practitioner perceptions, particularly considering the documented efficacy of telehealth interventions. To advance the field, future research should include longitudinal studies that examine the long-term outcomes of telehealth interventions, explore hybrid models of service delivery, and assess specific factors that may influence success, such as patient preferences and technological competence (Robinson et al., 2021). While the AOTA has advocated for Medicare and Medicaid reimbursement for telehealth services, which would help solidify its role in practice, it is essential to position telehealth not only as an emergency response tool but as a valuable and routine service delivery model that can expand access to care and improve occupational outcomes (Proffitt et al., 2021). These efforts would ensure telehealth's continued role as an essential part of occupational therapy practice, well beyond public health crises.
